# Association of *GABRG2* gene polymorphisms with idiopathic generalized epilepsy in Egyptian children: a case–control study

**DOI:** 10.1186/s40001-025-03230-x

**Published:** 2025-10-27

**Authors:** Yahya Wahba, Doaa Shahin, Abdel-Hady El-Gilany, Sohier Yahia, Nora El-Tantawy, Mohamed Khodair, Hanan Sherbiny, Hanaa Khalil, Khadeja Soheeb

**Affiliations:** 1 Department of Pediatrics, Faculty of Medicine, Mansoura University, Mansoura, Egypt; 2 Clinical Haematology Unit, Department of Clinical Pathology, Faculty of Medicine, Mansoura University, Mansoura, Egypt; 3 Department of Public Health, Faculty of Medicine, Mansoura University, Mansoura, Egypt; 4 Department of Public Health, Applied Medical Sciences Faculty, Al-Baha University, Al-Baha, Saudi Arabia; 5 Department of Parasitology, Faculty of Medicine, Mansoura University, Mansoura, Egypt; 6 Department of Neuropsychiatry, Faculty of Medicine, October 6 University, Giza, Egypt; 7 Department of Child Health, Faculty of Medicine, Bisha University, Bisha, Saudi Arabia; 8 Department of Pediatrics, Elmam AbdElRhman ElFaisal Hospital, Riyadh, Saudi Arabia; 9 Department of Pediatrics, Faculty of Medicine, Zawia University, Zawia, Libya

**Keywords:** *GABRG2* 3145G>A, *GABRG2* C588T, Idiopathic generalized epilepsy, Polymorphism

## Abstract

**Purpose:**

The *GABRG2* gene polymorphisms C588T and 3145G>A could be predictive genetic markers that trigger idiopathic generalized epilepsy (IGE) or predict pharmaco-resistance to antiseizure medications (ASMs).

**Methods:**

This case‒control study enrolled 85 children, including 34 patients with IGE who were responsive to ASMs (responsive group), 30 patients with IGE who were resistant to ASMs (resistant group), and 21 healthy children (control group). All participants were assessed for the *GABRG2* C588T and *GABRG2* 3145G>A gene polymorphisms via polymerase chain reaction (PCR).

**Results:**

The CC genotype of the *GABRG2* polymorphism was the most commonly reported genotype. The CT and TT genotypes were more frequently associated with epileptic patients than with controls. The T allele and the T-included genotypes were more common among epileptic patients than controls. Regarding the *GABRG2* _3145G>A polymorphism, the AG genotype was the most frequent among the study groups. The GG phenotype was more common among epileptic children than in controls. The G allele and G-included genotypes were significantly associated with epilepsy (*p* = 0.02), with a 3.2-fold higher risk of occurrence of epilepsy for the G allele carriers. A statistically insignificant difference in the distribution of different genotypes and C & T alleles of the *GABRG2* C588T polymorphism was detected between the ASMs-responsive and the ASMs-resistant subgroups. However, the TT genotype was more common in the ASMs-resistant subgroup (10% vs. 3%). The *GABRG2*_3145G>A polymorphism appeared to be a prognostic determinant of ASMs responsiveness; the GG genotype was significantly associated with poor control of seizure activity (47% vs. 24%, *p* = 0.05). The G-included genotypes were significantly associated with ASMs resistance (76% vs. 53%, *p* = 0.05).

**Conclusions:**

The T allele and TT genotype of the *GABRG2* C588T gene were more common among patients with IGE, whereas the G allele and the GG genotype of the *GABRG2* 3145G>A gene may be significant predictors of ASMs resistance among IGE patients. Results validation in larger, multi-center studies across diverse populations is recommended.

## Background

Epilepsy is a frequent disorder with complex inheritance affecting an estimated 10.5 million children globally, representing a significant global health burden [1]. Idiopathic generalized epilepsy (IGE) comprises approximately one-third of all epilepsy disorders [2]. This type of epilepsy is defined by recurrent generalized seizures in individuals with normal development and no neurological impairment or brain structural lesions [3]. Although the pathogenesis of epilepsy is still vague, there is growing evidence that genetic factors are crucial for both the selection and the efficacy of antiseizure medications (ASMs) in pediatric patients [4–6]. Genetic variations usually affect channel functions, changing electrical impulses by modifying neuronal excitability and promoting a network of neurons into a synchronous activity, which can result in epileptic seizures [7].

The primary inhibitory neurotransmitter in the brain, gamma-amino butyric acid (GABA), acts via two classes of specific receptors, GABA_A_ and GABA_B_ [8]_._ The most commonly found receptor in the human central nervous system (CNS) is the GABA_A_ receptor, which functions primarily as a hetero-oligomer pentamer with *α*, *β*, and either *γ* or *δ* subunits. As a result, it has been suggested that IGE is largely caused by GABA_A_ receptor dysfunction [9]. Genetic variation in the genes encoding GABA_A_ receptor subunits, specifically GABAR-A_1_, GABAR-B_2_, and GABAR-G_2,_ is highly associated with the pathogenesis of IGE [10].

The *GABRG2* gene encodes the ɣ2 subunit of the GABA_A_ receptor, which is known to change the kinetics of GABA_A_ receptors by altering channel functions, synaptic and postsynaptic clustering, and maintenance [11]. Wang et al. described *GABRG2* gene polymorphism as a modulating factor of gene expression with subsequent aberrant ɣ2 subunit conformation or function [12]. Moreover, GABA_A_ receptors are the molecular target of many ASMs, either by direct agonistic action or by boosting the level of GABA [8]. The barbiturate, diazepam and topiramate are common examples of drugs that interact with GABA_A_ receptors [12]. Recently, associations were established between the phenotype of patients with *GABRG2* genetic variants and the functional consequences of these variants on GABAA receptor activity [13].

Single nucleotide polymorphisms (SNPs) of the *GABRG2* gene have been assumed to be potential risk factors for different types of seizures and are potential genetic determinants of pharmacological resistance to ASMs among different ethnic populations; however, unfortunately, the results are inconclusive and occasionally contradictory [4–6], [8].

*GABRG2* C588T polymorphism is one of the most commonly studied SNPs of the *GABAG2* gene among patients with IGE but with conflicting results regarding the disease association and response to ASMs [4, 5, 12]. *GABRG2* 3145G>A is another SNP with a possible association with IGE but with limited studies [14, 15]

The current study evaluated two polymorphisms of the *GABRG2* gene (*GABRG2* C588T and *GABRG2* 3145G>A) as risk predictors for IGE and their role as prognostic factors of resistance to ASMs.

## Methods

### Study design and participants

This observational case‒control study was conducted at Mansoura University Children's Hospital, Egypt, from September 2022 to September 2024. There were intervals when participant recruitment was paused. Sixty-four children with IGE represented the case group and were subdivided into antiseizure medications-responsive (34 ASMs-responsive) and antiseizure medications-resistant (30 ASMs-resistant) subgroups according to their response to standard ASMs during the last year. Twenty-one healthy children of the same ethnicity and residency were recruited from general pediatric outpatient clinics of the same hospital as the control group. The age and sex of the patients were comparable to those of the case groups. Controls were recruited while visiting the hospital for mild illnesses such as upper respiratory tract infection or gastroenteritis, and none of them had a family or personal history of CNS insult or seizure disorders.

Each participant had a complete medical history, with particular regard to bio-demographic data, clinical data (seizure type, ASMs type and dose, duration, drug response, frequency, and seizure duration, time of first and last seizures), and full neurological and general examinations were performed. The relevant patient data were confirmed from each patient's medical file. The results of the electroencephalography (EEG) and brain magnetic resonance imaging (MRI) were also retrieved from patients’ files. The seizure type was assessed based on the 2017 International League Against Epilepsy (ILAE) operational classification [16]. The clinical diagnosis of epilepsy, whether focal or generalized, was confirmed by EEG findings [17].

Patients with IGE were defined as patients with a generalized epileptic onset having normal interictal neurological and developmental milestones, with no recognized neurological insults either clinically or radiologically, namely, by brain MRI [18]. They mostly have positive generalized spikes in their EEG signals. Generalized seizures include childhood absence epilepsy, juvenile absence epilepsy, juvenile myoclonic epilepsy, and primary generalized tonic‒clonic seizures [3]. If the patient had not experienced any seizures for at least one year, they were considered ASMs-responsive; if two tolerated, properly chosen, and administered ASMs had failed to achieve sustained freedom from seizures, they were categorized as ASMs-resistant. [19].

Patients who had experienced two or more unprovoked seizures in the past 24 h, normal neurological development and examination results, normal brain MRI findings, and generalized bilateral synchronous EEG findings were included in the research. The study excluded children with a diagnosis of uncertain epilepsy, metabolic disorders, secondary epilepsies, psychiatric disorders, uncompliant patients, patients with atypical convulsions (complex febrile seizures), patients with focal seizures, and patients with cerebral anomalies detected by brain MRI. Before being included in the research investigation, every participant's legal guardian provided written informed consent. Mansoura University's Faculty of Medicine Institutional Review Board (IRB) acknowledged the entire study design. Personal privacy and confidentiality were respected throughout the entire study. All methods were performed per the relevant guidelines and regulations.

### Sample size ***calculation*** [20]

The OSSE online sample size estimator, accessible at osse.bii.a-star.edu.sg, was used to determine the sample size [20]. Butilă et al. [15] determined the sample size on the basis of their findings of a (38%) variation in the minor frequency allele (T) of the *GABRG2* C588T gene polymorphism between IGE patients who were ASMs resistant and those who were ASMs responsive. The study’s power was 80%, and the significance level was 5%. At least 30 patients per group were included in the sample. Applying the Hardy‒Weinberg model to the available data facilitated the calculation of the genotype frequency deviation from Hardy‒Weinberg equilibrium.

### Blood samples and laboratory investigations

Under completely aseptic conditions, 5 ml of blood were obtained from every participant, and 2 ml was kept on EDTA for complete blood count (CBC) and for genetic assessment of alleles and genotyping of *GABRG2* C588T and *GABRG2* G3145A gene polymorphisms via PCR and restriction fragment length polymorphism (RFLP) via restriction enzymes. The remaining 3 ml was used for routine liver and kidney function tests, including assessments of SGOT, SGPT and creatinine.

### Genotyping for GABRG2 C588T and GABRG2_3145 > A gene polymorphisms

**Fig. 1:**
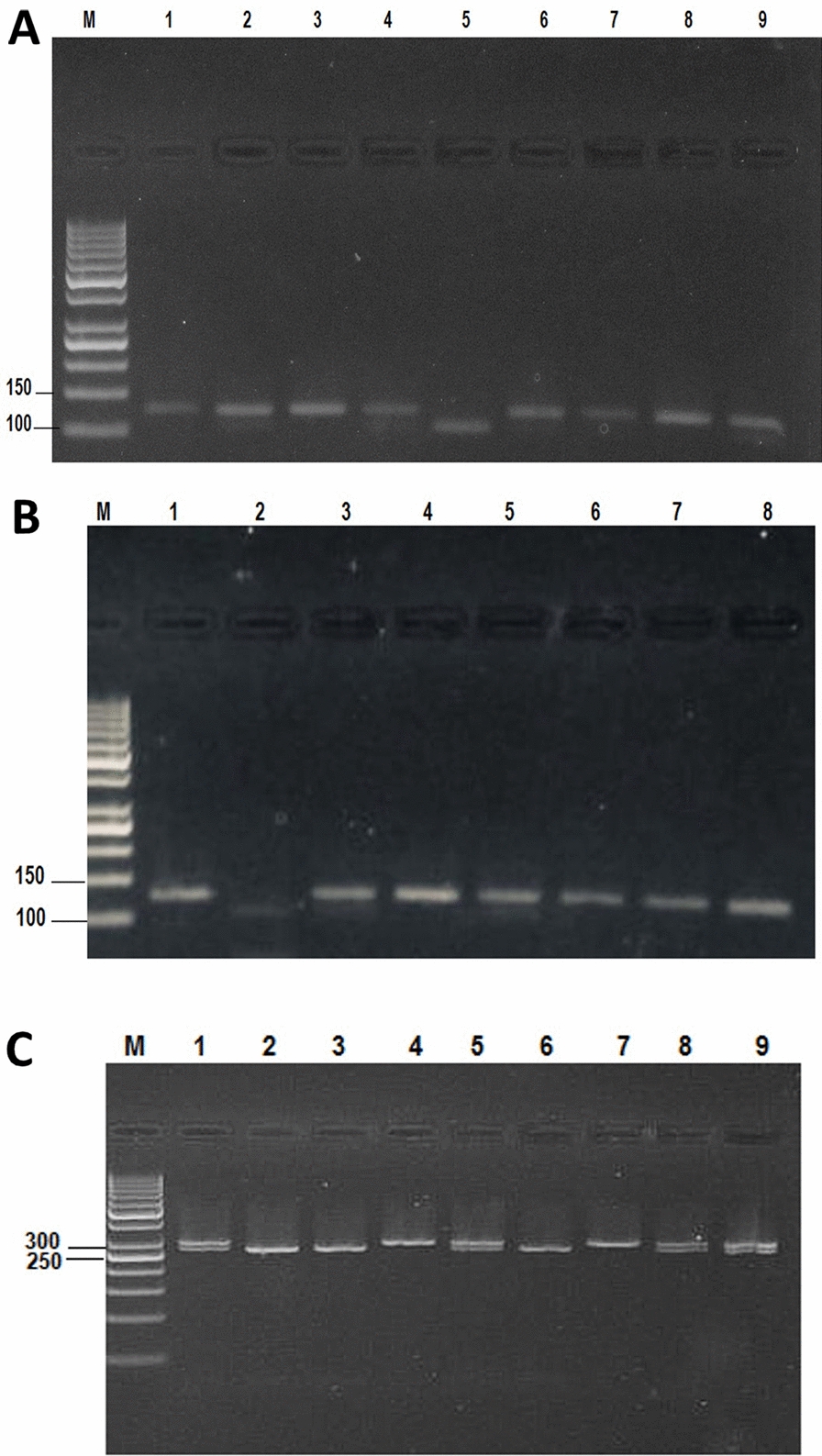
**A** Polymerase chain reaction product of *GABRG2 C.588 T* by the *ApoI* enzyme. M: 50 bp marker; Lanes 1, 3, 7, and 8 are CC alleles at 122 bp; Lanes 2, 4, 6, and 9 are CT alleles at (100 bp and 122 bp); Lane 5 is the TT allele at 100 bp. **B** Agarose electrophoresis after enzyme digestion of *GABRG2* C.588 T by the *ApoI* enzyme. M: 50 bp marker; Lanes 1, 4, 6, 7, and 8 are CC alleles at 122 bp; Lanes 3 and 5 are CT alleles at (100 bp and 122 bp); Lane 2 is the TT allele at 100 bp; and **C** agarose electrophoresis after enzyme digestion of *GABRG2* 3145 G > A by the *Ncil* enzyme. M: 50 bp marker; Lanes 1, 5, 8, and 9 are AG alleles at (330 bp and 270 bp); Lanes 2, 3, and 6 are GG alleles at 270 bp; Lanes 4 and 7 are AA alleles at 330b

Employing Generation DNA Purification capture column kits (Fermentas, USA), genomic DNA was extracted from 200 µL of whole blood. Electrophoresis employing a 1% agarose gel was used to detect the extracted DNA. The gel containing the DNA samples was immersed in 1 × Tris–phosphate-EDTA buffer (TPE) and electrophoresed for 20 min at 200 V. Ethidium bromide staining was used to visualize the DNA, and a digital camera was used to obtain photographs of the gel (Fig. 1A).

A single-tube tetra-primer polymerase chain reaction (PCR) assay was utilized to detect and amplify the *GABRG2* C588T and *GABRG2* 3145G>A gene polymorphisms. The cycling conditions included initial denaturation for 5 min at 95 °C, followed by 37 cycles (for *GABRG2* 3145 G>A) or 35 cycles (for *GABRG2* C588T) of denaturation for 30 s at 94 °C, primer attachment (annealing) for 30 s at 60 °C for *GABRG2* 3145G>A and 55 °C for *GABRG2* C588T, and then extension for 45 s at 72 °C, with a final extension for 7 min at 72 °C in both cases. The sequencing primer (forward primer: 5′-GAG TGC CAA TTA CAA TTG CAA AA-3′; reverse primer: 5′-AAT CAG AAA GAC TGT AGG TGA GG-3′) was used for detection of the *GABRG2* C588T gene polymorphism. The sequencing primer (forward primer: 5′-AGA AAT TTA CCA ACT GGT CTA GCC GG-3′; reverse primer: 5′-AAA TCA AAT ATT GTG TCA TGC TTA GT-3′) was used for detection of the *GABRG2* 3145G>A gene polymorphism [14]. *Apol* and *Ncil* restriction enzymes were used for the detection of the *GABRG2* C588T and G3145A polymorphisms, respectively. The detection of digested products via gel electrophoresis depends on the idea that DNA fragments migrate at different speeds on the basis of their weight [21]. Bands of CC, CT, and TT appeared at 122 bp, 100 bp, and 122 bp and 100 bp, respectively **(**Fig. 1B**)**. Bands of the (AG), (GG), and (AA) genotypes appeared at 330 bp and 270 bp, 270 bp and 330 bp, respectively **(**Fig. 1C**)**.

### Statistical analysis

SPSS version 26 (IBM Corp, Armonk, NY) was used to analyze the data. Fisher’s exact and the chi-square tests were used to compare the qualitative data, which were presented as percentages and numbers. Following a Kolmogorov‒Smirnov test for normality, quantitative data were reported as the means ± SDs for parametric data and medians (minimum‒maximum) for non-parametric data. Parametric quantitative data from two groups were compared via Student’s *t* test, whereas non-parametric quantitative data from two groups were analyzed via the Mann‒Whitney test. The analysis of parametric quantitative data from more than two groups was performed via one-way ANOVA. We presumed statistical significance at *p* ≤ 0.05. The odds ratio (OR) and 95% confidence interval (CI) of the odds ratio were calculated to estimate the risk of association between polymorphism genotypes and different alleles among patients and controls.

## Results

The demographic, clinical and EEG findings of the enrolled subgroups were summarized and tabulated (Table 1). The cases and controls were matched regarding the age, gender, family history of seizure and positive consanguinity (*p* > 0.05). Male sex was not significantly different between epileptic and healthy peers, who were intentionally chosen to be comparable with the epileptic group; however, when ASMs-responsive and ASMs-resistant subgroups were compared, significantly more boys were included in the ASMs-responsive population (*p* = 0.003). Regardless of ASMs responsiveness, tonic‒clonic seizures were the most common presenting seizure type among all epileptic patients (64.7% ASMs-responsive patients vs. 76.7% ASMs-resistant patients, *p* = 0.3). The second most common presenting epileptic type was tonic seizures (20.6%) in the ASMs-responsive subgroup and absence seizures (10%) in the ASMs-resistant subgroup. Valproic acid and levetiracetam were the most prescribed ASMs in both groups, with no statistically significant difference in their use between the ASMs-responsive and ASMs-resistant groups. Carbamazepine was used more frequently for ASMs-responsive patients (20.6%), whereas ethosuximide was used for treating absence seizures (8% and 10% in ASMs-responsive and resistant subgroups, respectively) either alone or in conjunction with other ASMs, whereas topiramate was a common add-on ASMs for the resistant group. A significantly greater number of normal EEGs were reported among ASMs-responsive patients at diagnosis than ASMs-resistant patients (*p* = 0.044).

**Table 1:** Bio-demographic, clinical, and electroencephalography (EEG) data of the study groups

	Control (*N* = 21)	ASMs-response epilepsy (*N* = 34)	ASMs-resistant epilepsy (*N* = 30)	Test	*p* value
Age (years) (mean ± SD)	8.9 ± 2.3	10.2 ± 4	9.5 ± 3.6	ANOVA	*p*1 = 0.18 *p*2 = 0.5 *p*3 = 0.5
Gender	10 (47.6%)	40 (62%)		*χ*^*2*^ 1.44	*p* = 0.2
Males, *N* (%)	10 (47.6%)	27 (79.4%)	13 (43.3%)	1 *χ*^*2*^ 5.88 2*χ*^*2*^ 0.08 3 *χ*^*2*^ 8.6	*p*1 = 0.001* *p*2 = 0.7 *p*3 = 0.003^*^
Females, *N* (%)	11 (52.4%)	7 (20.6%)	17 (56.7%)		
Positive family history, *N* (%)	0	10 (29.4)	14 (48.7)	*χ*^*2*^ = 2.4	*p*1 NA *p*2 NA *p*3 = 0.12
Positive consanguinity, *N* (%)	9 (42.9)	8 (23.5)	6 (20)	1 *χ*^*2*^ 2.18 2 *χ*^*2*^ 4.12 3 *χ*^*2*^ 0.08	*p*1 = 0.13 *p*2 = 0.09 *p*3 = 0.7
Weight (mean ± SD)	32.8 ± 10.4	36.4 ± 18.3	31.6 ± 12.5	t student 0.93 0.7 0.91	*p*1 = 0.41 *p*2 = 0.7 *p*3 = 0.2
Seizure types					
GTC					
Tonic‒clonic		22 (64.7)	23 (76.7)	*χ*^*2*^ = 1.06	*p*3 = 0.3
Tonic		7 (20.6)	2 (6.7)		
Clonic		2 (5.9)	–		
Juvenile myoclonic epilepsy (JME)		–	2 (6.6)	*χ*^*2*^ = 2.21	*p*3 = 0.13
Childhood and juvenile absence epilepsy		3 (8.8)	3 (10)	*χ*^*2*^ = 0.077	*p*3 = 0.7
Age first onset (months) [median (min–max]		33 (6–132)	30 (1–120)	*t^* = 0.96	0.9
Last seizure (months) [median (min–max]		24 (13–60)	1 (1–6)	*t^* = 0.7	≤ 0.001^*^
EEG findings					
Normal		15 (44.4)	6 (20)	*χ*^*2*^ = 4.1	0.044^*^
Centrocephalic (GSWDs)		19 (55.9)	24 (80)	*χ*^*2*^ = 3.03	0.08
Antiseizure medications (ASMs)					
Valproic acid		28 (82.4)	27 (90.9)	*χ*^*2*^ = 0.55	0.4
Levetiracetam		9 (26.5)	13 (43.3)	*χ*^*2*^ = 0.21	0.2
Carbamazepine		7 (20.6)	4 (13.3)	*χ*^*2*^ = 0.55	0.4
Oxcarbazepine		2 (5.9)	2 (6.7)	*χ*^*2*^ = 0.09	0.9
Topiramate		1 (2.9)	3 (10)	*χ*^*2*^ = 1.3	0.2
Ethosuximide		3 (8)	3 (10)	*χ*^*2*^ = 0.04	0.9

*GTC* epilepsy with generalized tonic–clonic seizures; *GSWDs* generalized spike-and-wave discharges

*p*1: difference between the control group and the ASMs-responsive epilepsy group; *p*2: difference between the control group and the ASMs-resistant epilepsy group; *p*3: difference between the ASMs-responsive group and the ASMs-resistant epilepsy group; *χ*^*2*^, chi-square test; Student’s *t* test; Student’s *t*-square test–Whitney test; ^*^ = statistically significant.

**Fig. 2:**
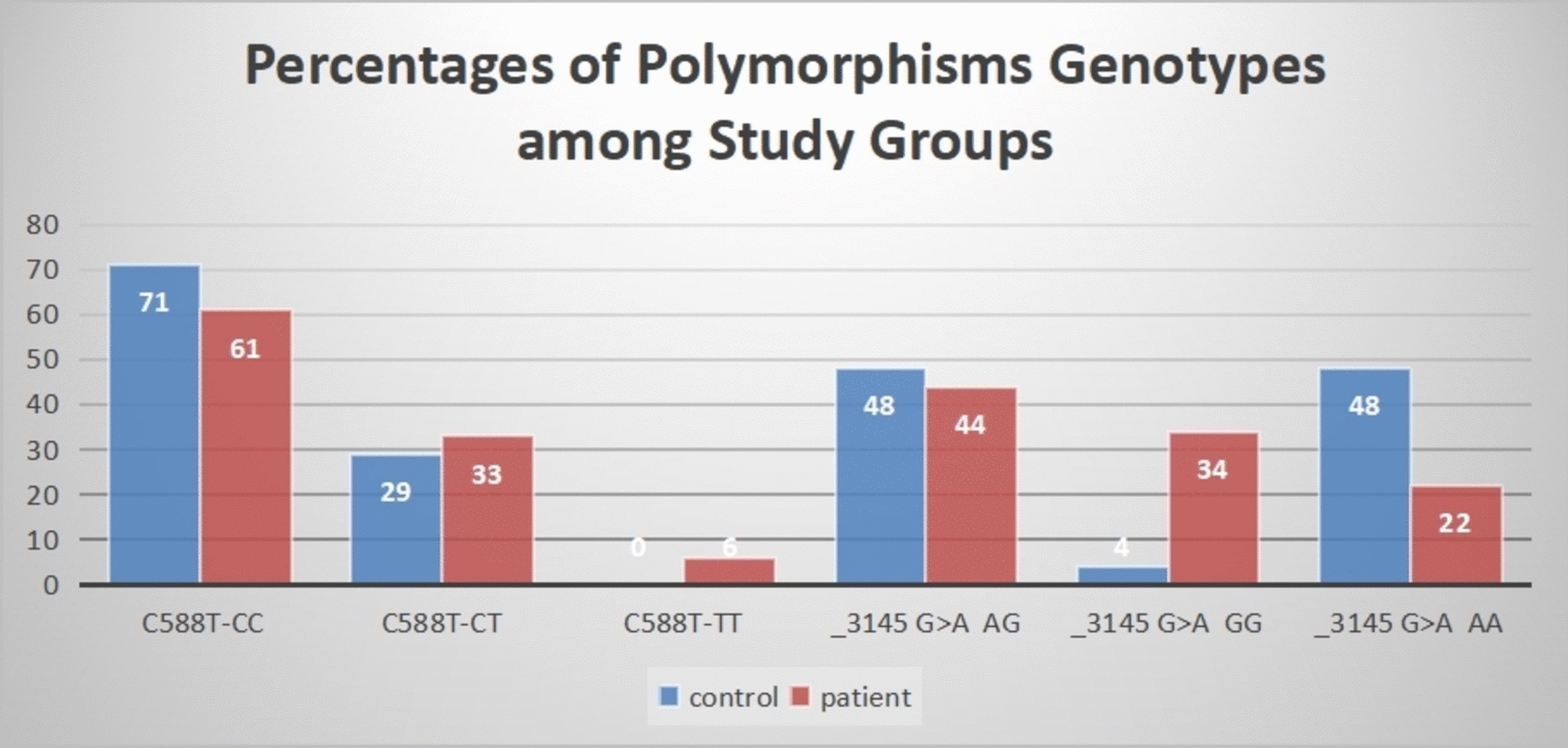
Percentages of *GABRG2* polymorphism genotypes among the study groups

Evaluating *GABRG2* polymorphisms (C588T and 3145G>A) as risk predictors for IGE among Egyptian children was our main goal. Comparisons of the findings between epileptic children and healthy controls are summarized in Table 2 and Fig. 2. Regarding *GABRG2* C588T polymorphism, the CC genotype was the most commonly reported genotype among all enrolled children (71% control vs. 61% epileptic patients, *p* = 0.4), and the CT and TT genotypes were more frequently associated with epileptic patients than controls (33% vs. 29% for CT and 6% vs. zero for TT). The C- included genotypes were displayed more frequently among controls than patients. In contrast, the T allele and T-included genotypes (39% vs. 29%) were more frequent among epileptic patients than among controls. However, the differences between the groups were not statistically significant, and a significant odds ratio for the association between the *GABRG2* C588T polymorphism and the development of IGE was not identified.

**Table 2:** *GABRG2* polymorphism genotypes and allele frequencies among the study groups

Group variables	Controls (21)	Patients (64)	Test *χ*^*2*^	*p* value	Odd ratio (CI) & *p*
*GABRG2* C588T polymorphism					
Genotypes, *N* (%)					
CC	15 (71)	39 (61)	0.67	0.4	
CT	6 (29)	21 (33)	0.18	0.67	
TT	0 (0)	4 (6)	1.3	1.3	
CC + CT/TT	21/0 (100)	60 (94)	1.3	0.25	0.3 (0.01–6.05) 0.44
CT + TT/CC	6/15 (29)	25 (39)	0.67	0.4	1.6 (0.54–4.6) 0.38
Alleles, *N* (%)					
C	36/42 (86)	99/128 (77)	1.54	0.21	0.56 (0.21–1.48) 0.24
T	6/42 (14)	29/128 (23)	1.54	0.21	1.75 (0.67–4.5) 0.24
*GABRG2*-3145G>A polymorphism					
Genotypes, *N* (%)					
AG	10 (48)	28 (44)	0.1	0.7	
GG	1 (4)	22 (34)	6.6	0.009^*^	
AA	10 (48)	14 (22)	4.8	0.02^*^	
AG + GG/AA	11/10 (52)	50/14 (78)	5.2	0.02^*^	3.2 (1.14–9.19) 0.02^*^
AG + AA/GG	20/1 (95)	40/24 (62)	8.1	0.004^*^	0.08(0.01–0.6) 0.01
Alleles, *N* (%)					
A	30 (71)	56 (44)	4.55	0.03	0.3(0.14–0.66)0.002
G	12 (29)	72 (56)	5.57	0.03	3.2 (1.51–6.8) 0.03^*^

^*^Statistically significant

Regarding *GABRG2* _3145G>A polymorphism, the AG genotype was the most frequently reported genotype among the study groups (48% vs. 44% for controls and patients, respectively). The GG phenotype was more common among epileptic children than controls (34% vs. 4% for patients and controls, respectively), whereas the reverse was observed for the AA genotype, which was significantly more common among controls (48% vs. 22% for controls and patients, respectively). The G allele and G-included genotypes were significantly associated with epilepsy (78% vs. 52%, *p* = 0.02), with a 3.2-fold higher risk of occurrence of epilepsy in the G allele carriers. The A allele and A-included genotypes were significantly associated with controls with a minimal risk for epilepsy (95% vs. 62%, *p* = 0.004).

The second main issue of interest is the associations between different *GABRG2* polymorphisms and the efficacy of ASMs. Data from the ASMs -responsive and ASMs-resistant subgroups are presented in Table 3. Insignificant differences in the distributions of different genotypes and C & T alleles of the *GABRG2* C588T polymorphism were detected between the ASMs-responsive and ASMs-resistant subgroups. However, the TT genotype was reported more frequently among the ASMs-resistant subgroup (10% vs. 3%). On the other hand, the *GABRG2* _3145G>A polymorphism appeared to be a prognostic determinant of ASMs responsiveness, and the GG genotype was significantly associated with poor control of seizure activity despite the well-chosen and adherent use of ≥ 2 ASMs (47% vs. 24%, *p* = 0.05). The G- included genotypes were significantly associated with ASMs resistance (76% vs. 53%, *p* = 0.05).

**Table 3:** *GABRG2* polymorphism genotypes and allele frequencies among epileptic groups

Group variable	ASMs-responsive (34)	ASMs-resistant (30)	Test *χ*^*2*^	*p* value	
*GABRG2* C588T polymorphism					
Genotypes, *N* (%)					
CC	20 (59)	19 (63)	0.10	0.7	1.2 (0.4–3.3)
CT	13 (38)	8 (27)	0.86	0.3	0.6 (0.2–1.7)
TT	1 (3)	3 (10)	1.30	0.25	3.7 (0.4–37.3)
CC + CT/TT	33/1 (97)	27/3 (90)	1.3	0.25	0.27 (0.02–2.775) 0.27
CT + TT/CC	14/20 (41)	11/19 (36)	0.16	0.6	0.8 (0.30–2.26) 0.71
Alleles, *N* (%)					
C	53/68 (78)	46/60 (77)	0.01	0.8	0.92(0.40–2.12) 0.86
T	15/68 (22)	14/60 (23)	0.01	0.8	1.075(0.46–2.46) 0.86
*GABRG2*-3145G>A polymorphism					
Genotypes, *N* (%)					
AG	20 (59)	8 (27)	6.5	0.01^*^	0.3(0.1–0.7)
GG	8 (24)	14 (47)	3.6	0.05^*^	2.8(1.0–8.3) 0.05^*^
AA	6 (17)	8 (26)	0.76	0.38	1.7(0.5–5.6)
AG + GG/AA	28/6 (82)	22/8 (73)	0.73	0.39	0.58(0.17–1.95) 0.3
AG + AA/GG	26/8 (76)	16/14 (53)	3.66	0.05^*^	0.35(0.12–1.02) 0.055
Alleles, *N* (%)					
A	32/68 (47)	24/60 (40)	0.63	0.42	0.75(0.37–1.52) 0.4
G	36/68 (53)	36/60 (60)	0.63	0.42	1.33 (0.6–2.69) 0.42

*ASMs* antiseizure medications

^*^Statistically significant

## Discussion

Gamma-amino butyric acid (GABA) is the master inhibitory neurotransmitter that counterbalances excitatory forces in the human CNS. GABA_A_ receptors are ligand-gated chloride channels that hyperpolarize neurons by increasing inward chloride conductance and initiating rapid inhibition of the postsynaptic potential [22]. The perturbation of the excitatory/inhibitory balance due to variations in *GABA* receptor gene sequences is the main theory behind monogenetic seizures [3] [23]. As the *GABRG2* gene encodes the ɣ2 subunit of GABA_A_ receptors, which is reported to be the crucial determinant of receptor function [11], and on the basis of recent studies that determined that the target action of many ASMs is GABA receptor-mediated [12] [24], we hypothesized that genetic polymorphisms of the *GABRG2* gene may influence the susceptibility of Egyptian children to IGE and may also impact ASMs effectiveness in controlling their seizure activity. Two *GABRG2 (GABRG2* C588T and *GABRG2* 3145G>A*)* gene polymorphisms were evaluated in the present study via PCR (RFLP) as IGE risk predictors and ASMs efficiency-determining factors.

Regarding the *GABRG2* C588T gene polymorphism, the C allele and C- included genotypes were more frequent among healthy children, whereas the T allele and T-included genotypes were more frequent among patients with epilepsy. However, the differences between groups did not reach statistical significance, and the odds ratio for the association between the *GABRG2* C588T polymorphism and the development of IGE was undetectable. Moreover, the distributions of different genotypes and C & T alleles of the *GABRG2* C588T polymorphism were not significantly different between the ASMs-responsive subgroup and the ASMs-resistant subgroup; nevertheless, the TT genotype was reported more frequently among the ASMs-resistant subgroup. Several previous studies from different ethnic groups, including a recent article conducted in a nearby Egyptian governorate, had evaluated the associations between the *GABRG2* C588T gene polymorphism and the seizure risk and/or ASMs efficacy, but they reported conflicting results [4, 14, 15, 25, 26]. The first study that investigated this association was conducted in Taiwan, and they reported that the *GABRG2* C588T-C allele was a significant risk factor for IGE [14]. In contrast, Balan and colleagues agreed with the initial finding of an association between the *GABRG2* C588T C allele and the CC genotype with mesial temporal lobe epilepsy and juvenile myoclonic epilepsy but could not find a real effect of different genotypes on the efficacy of ASMs [5]. On the other hand, an earlier Indian study reported the predominance of the *GABRG2* C588T-T allele and TT genotype among children with IGE, particularly those with poor control with ASMs [4]. Another Indian study reported a lack of significant associations between the *GABRG2* C588T polymorphism and IGE [26]. The screening of Romanian children with IGE for the *GABRG2* C588T gene polymorphism revealed a strong association of the *GABRG2* C588T-T allele and TT genotype with IGE risk and pharmaco-resistance to standard ASMs [15]. Disputing this association, several previous studies from Brazil, India, and Germany did not reveal a significant association between the *GABRG2* C588T T allele frequency or TT genotype and the risk of IGE or the failure of ASMs therapy [26–28]. A previous Egyptian study documented the link between the *GABRG2* C588T-T polymorphism and the risk of developing IGE and pointed to carrying the T allele and TT genotype as poor prognostic factors for ASMs responsiveness [25].

The puzzled results of the aforementioned studies are not surprising in the genetic world, where topographic variation with different ethnic origins is a logical rationale for the different genetic distributions of *GABRG2* C588T polymorphisms [4, 26, 29, 30] that lead to differences in gene expression [12] and associated differences in encoding receptor unit conformations [31]. Wang et al. [12] described the prevalence of the T allele and TT genotype as “population-specific” polymorphisms. Variable impaired pathways of the mutant *GABRG2* gene, such as mRNA instability, atypical subunit folding, distorted GABA-gated chloride channels, and defective glycosylation, lead to variation in GABA_A_ receptor assembly with minimal surface expression and response to external effects [32]. Occasionally, functional loss of GABA_A_ receptors by a decrease in the level of GABA expression or the acceleration of its deactivation may be the end result of different *GABA* polymorphisms [33]. Notably, the combined results of meta-analysis and expression quantitative trait locus (QTL) analysis via the BRAINEAC database revealed that the *GABRG2* C588T polymorphism was associated with IGE under dominant and allelic models among Asian populations but not among Caucasians [12].

Wide-spectrum discrepancies in published results from the same locality and ethnic background, as observed in Indian and Egyptian publications [4, 5, 25, 26], cannot be explained by ethnic variability alone but can be reasonably attributed to other confounding factors that affect gene expression, including epigenetic effects, environmental stressors, and different levels of counterbalancing excitatory forces [12, 22].

Epigenetic alteration can affect gene expression by modifying chromatin without affecting the DNA sequence, with subsequent gene activation or suppression [33]. Finally, *GABRG2* C588T polymorphisms influence GABAG2 expression in selective areas of the human brain, which in turn affects GABA levels in certain areas and not the entire CNS, leading to different aberrant electrical activities [12].

Conflicting results concerning the prognostic role of the *GABRG2* C588T polymorphism on ASMs efficacy have been reported when the almost absent effect among our cohort was contrasted with the highly effective impact of the T allele and TT genotype on ASMs pharmaco-resistance documented by different studies [12, 25, 29]. An association between the central GABA level and its receptors and ASMs effectiveness has been proposed and illustrated: benzodiazepine and barbiturate enhance and prolong the binding of GABA with its receptors, whereas vigabatrin and tiagabine increase the synaptic GABA level by inhibiting its metabolism and reuptake [24]. Moreover, proteins encoded by *GABA* genes interact with several ASMs [12].

Studies have described polymorphisms of the *GABA* genes as important risk factors for ASMs resistance and revealed the potentiality of these genes as therapeutic targets for refractory epilepsy [12, 15, 25, 34]. Failure to prove such an association among our cohort can be explained by several factors, including the small size of our ASMs resistant group (30 patients), which might distort the final results. Studying the effects of gene polymorphisms is a costly technique at the personal level and requires the initiative of multiple institutes to involve mega samples and obtain more controlled results [12, 15]. Other factors that may explain the antagonistic effects of the *GABRG2* C588T polymorphism on ASMs efficacy are the wide variability in patient characteristics (patient phenotypes), the variation in ASMs protocols and the flexibility of the definition of ASMs resistance at different medical institutes [12, 25].

Regarding the polymorphism at nucleotide position 3145 in intron G > A (*GABRG2*_3145G>A polymorphism), the AG genotype was the most frequently reported genotype among the study participants. The presence of the G allele was associated with a threefold higher risk of epilepsy, and the GG phenotype has a higher frequency among epileptic children with a doubled chance of ASMs resistance. Our results appeared to be unique in this area, as the *GABRG2*_3145G>A polymorphism has rarely been studied in previous studies and was uniformly described as insignificant risk factor for IGE, febrile seizure or ASMs resistance [14, 15]. The AG genotype was the most frequently detected genotype, and the loss of heterogeneity in the GG genotype was recognized to be associated with IGE cases and more frequently reported among the ASMs-resistant subgroup. Chou and his team were the first to assess the intronic SNP as a risk factor for IGE; according to previous literature, the AA genotype was used as a reference, and they could not find any association between the *GABRG2*_3145G>A polymorphism and the risk of IGE [14]. A Romanian study evaluating the value of the *GABRG2*_3145G>A polymorphism as a predictor of IGE and/or febrile seizure failed to find any statistically significant difference between patients with IGE or febrile seizure and healthy controls [15]. In contrast, they reported that the AG genotype was a statistically frequent genotype among epileptic children, whereas the G allele fostering children presented a minimal risk of developing epilepsy. Genetic studies from different populations and different ethnic backgrounds have yielded contradictory results [12].

The strength of this study is the use of a clearly defined case–control design with well-characterized clinical subgroups of ASM-responsive and ASM-resistant IGE patients. The use of PCR-based genotyping ensured reliable detection of the targeted *GABRG2* polymorphisms with high analytical sensitivity and specificity. The study also examined both disease susceptibility and potential genetic predictors of drug resistance, which adds clinical relevance.

Study limitations: The relatively small sample size limits the statistical power and the ability to detect weaker associations. This limitation was primarily due to funding constraints, which restricted the number of participants who could be recruited and genotyped. Recruitment from a single center may further reduce the generalization of the findings.

## Conclusion

The T allele and TT genotype of the *GABRG2* C588T gene are more common among patients with IGE, whereas the G allele and GG genotype of the *GABRG2 3145G*>*A* gene may be significant risk factors for IGE occurrence and ASMs resistance among Egyptian IGE children. Our results support the opinion that the list of *GABA* gene sequence variations, polymorphisms and mutations associated with epilepsy are expected to grow further and that more SNPs are still waiting for detection and practical involvement as practical diagnostic and prognostic determinants of epilepsy. Multi-center studies with larger sample sizes, adequate funding and broader genetic profiling are needed to validate and extend these observations.

## Data Availability

All data generated or analyzed during this study are included in this published article.

## References

[1] Biset G, Abebaw N, Gebeyehu NA, Estifanos N, Birrie E, Tegegne KD. Prevalence, incidence, and trends of epilepsy among children and adolescents in Africa: a systematic review and meta-analysis. BMC Public Health. 2024;24:771. 38475724 10.1186/s12889-024-18236-z PMC10935902

[2] Devinsky O, Elder C, Sivathamboo S, Scheffer IE, Koepp MJ. Idiopathic generalized epilepsy: misunderstandings, challenges, and opportunities. Neurology. 2024;102:e208076. 38165295 10.1212/WNL.0000000000208076 PMC11097769

[3] Cossette P, Rouleau GA. Mutated GABA_A_ receptor subunits in idiopathic generalized epilepsy. Epilepsia. 2010;51:62–62. 22787675

[4] Ponnala S, Chaudhari JR, Jaleel MA, Bhiladvala D, Kaipa PR, Das UN, et al. Role of MDR1 C3435T and *GABRG2* C588T gene polymorphisms in seizure occurrence and MDR1 effect on anti-epileptic drug (phenytoin) absorption. Genet Test Mol Biomarkers. 2012;16:550–7. 22239287 10.1089/gtmb.2011.0225

[5] Balan S, Sathyan S, Radha SK, Joseph V, Radhakrishnan K, Banerjee M. *GABRG2*, rs211037 is associated with epilepsy susceptibility, but not with antiepileptic drug resistance and febrile seizures. Pharmacogenet Genomics. 2013;23:605–10. 24061200 10.1097/FPC.0000000000000000

[6] Haerian BS, Baum L, Kwan P, Cherny SS, Shin J-G, Kim SE, et al. Contribution of GABRG2 polymorphisms to risk of epilepsy and febrile seizure: a multicenter cohort study and meta-analysis. Mol. Neurobiol. 2016;53:5457–5467 10.1007/s12035-015-9457-y 26452361

[7] Hedrich U, Maljevic S, Lerche H. Mechanisms of genetic epilepsies. e-Neuroforum. 2013;4:23–30.

[8] Yang Y, Niu X, Cheng M, Zeng Q, Deng J, Tian X, et al. Phenotypic spectrum and prognosis of epilepsy patients with *GABRG2* variants. Front Mol Neurosci. 2022;15:809163. 35359574 10.3389/fnmol.2022.809163 PMC8964129

[9] Bhat MA, Guru SA, Mir R, Waza AA, Zuberi M, Sumi MP, et al. Association of GABAA receptor gene with epilepsy syndromes. J Mol Neurosci. 2018;65:141–53. 29785705 10.1007/s12031-018-1081-7

[10] Bhat MA, Guru SA, Mir R, Waza AA, Zuberi M, Sumi MP, et al. Association of GABAA receptor gene with epilepsy syndromes. J Mol Neurosci. 2018;65:141–53. 29785705 10.1007/s12031-018-1081-7

[11] Schweizer C, Balsiger S, Bluethmann H, Mansuy IM, Fritschy J-M, Mohler H, et al. The γ2 subunit of GABAA receptors is required for maintenance of receptors at mature synapses. Mol Cell Neurosci. 2003;24:442–50. 14572465 10.1016/s1044-7431(03)00202-1

[12] Wang S, Zhang X, Zhou L, Wu Q, Han Y. Analysis of *GABRG2* C588T polymorphism in genetic epilepsy and evaluation of *GABRG2* in drug treatment. Clin Transl Sci. 2021;14:1725–33. 33650258 10.1111/cts.12997 PMC8504831

[13] Rossi A, Lin SX, Absalom NL, Ortiz-De la Rosa S, Liao VW, Mohammadi NA, et al. Phenotypic Spectrum in Individuals With Pathogenic GABRG2 Loss- and Gain-of-Function Variants. Neurol. 2025;105(2):e213644 10.1212/WNL.0000000000213644 PMC12202131 40570274

[14] Chou I-C, Lee C-C, Tsai C-H, Tsai Y, Wan L, Hsu Y-A, et al. Association of *GABRG2* polymorphisms with idiopathic generalized epilepsy. Pediatr Neurol. 2007;36:40–4. 17162195 10.1016/j.pediatrneurol.2006.09.011

[15] Butilă AT, Zazgyva A, Sin AI, Szabo ER, Tilinca MC. *GABRG2* C588T gene polymorphisms might be a predictive genetic marker of febrile seizures and generalized recurrent seizures: a case-control study in a Romanian pediatric population. Arch Med Sci. 2018;14:157–66. 29379546 10.5114/aoms.2016.63739 PMC5778423

[16] Fisher RS, Cross JH, French JA, Higurashi N, Hirsch E, Jansen FE, et al. Operational classification of seizure types by the international league against epilepsy: position paper of the ILAE commission for classification and terminology. Epilepsia. 2017;58:522–30. 28276060 10.1111/epi.13670

[17] Scheffer IE, Berkovic S, Capovilla G, Connolly MB, French J, Guilhoto L, et al. ILAE classification of the epilepsies: position paper of the ILAE commission for classification and terminology. Epilepsia. 2017;58:512–21. 28276062 10.1111/epi.13709 PMC5386840

[18] Wyllie E, Gupta A, Lachhwani DK, editors. The treatment of epilepsy: principles & practice. Lippincott Williams & Wilkins; Philadelphia, USA. 2006.

[19] Kwan P, Arzimanoglou A, Berg AT, Brodie MJ, Allen Hauser W, Mathern G, et al. Definition of drug resistant epilepsy: consensus proposal by the ad hoc Task Force of the ILAE Commission on therapeutic strategies. Epilepsia. 2010. 10.1111/j.1528-1167.2009.02397.x. 19889013 10.1111/j.1528-1167.2009.02397.x

[20] Serdar CC, Cihan M, Yücel D, Serdar MA. Sample size, power and effect size revisited: simplified and practical approaches in pre-clinical, clinical and laboratory studies. Biochemia medica. 2021 Feb 15;31(1):27-53 10.11613/BM.2021.010502 PMC7745163 33380887

[21] Heller C. Principles of DNA separation with capillary electrophoresis. Electrophoresis. 2001;22:629–43. 11296917 10.1002/1522-2683(200102)22:4<629::AID-ELPS629>3.0.CO;2-S

[22] Treiman DM. GABAergic mechanisms in epilepsy. Epilepsia. 2001;42:8–12. 11520315 10.1046/j.1528-1157.2001.042suppl.3008.x

[23] Ellis CA, Petrovski S, Berkovic SF. Epilepsy genetics: clinical impacts and biological insights. Lancet Neurol. 2020;19:93–100. 31494011 10.1016/S1474-4422(19)30269-8

[24] The International League Against Epilepsy Consortium on Complex Epilepsies. Genome-wide mega-analysis identifies 16 loci and highlights diverse biological mechanisms in the common epilepsies. Nat Commun. 2018;9:5269. doi:10.1038/s41467-018-07524-z 10.1038/s41467-018-07524-z PMC6288131 30531953

[25] Abou El Ella SS, Tawfik MA, El Fotoh WMMA, Soliman OAM. The genetic variant “C588T” of GABARG2 is linked to childhood idiopathic generalized epilepsy and resistance to antiepileptic drugs. Seizure. 2018;60:39–43. 29894917 10.1016/j.seizure.2018.06.004

[26] Kumari R, Lakhan R, Kalita J, Misra U, Mittal B. Association of alpha subunit of GABAA receptor subtype gene polymorphisms with epilepsy susceptibility and drug resistance in north Indian population. Seizure. 2010;19:237–41. 20356767 10.1016/j.seizure.2010.02.009

[27] Gitaí LLG, de Almeida DH, Born JPL, Gameleira FT, de Andrade TG, Machado LC, et al. Lack of association between rs211037 of the *GABRG2* gene and juvenile myoclonic epilepsy in Brazilian population. Neurol India. 2012;60:585–8. 23287319 10.4103/0028-3886.105191

[28] Kananura C, Haug K, Sander T, Runge U, Gu W, Hallmann K, et al. A splice-site mutation in *GABRG2* associated with childhood absence epilepsy and febrile convulsions. Arch Neurol. 2002;59:1137–41. 12117362 10.1001/archneur.59.7.1137

[29] El-Beshlawy A, Salama AA, El-Masry MR, El Husseiny NM, Abdelhameed AM. A study of red blood cell alloimmunization and autoimmunization among 200 multitransfused Egyptian β thalassemia patients. Sci Rep. 2020;10:21079. 33273689 10.1038/s41598-020-78333-y PMC7713136

[30] Nakayama J, Hamano K, Noguchi E, Horiuchi Y, Iwasaki N, Ohta M, et al. Failure to find causal mutations in the GABAA-receptor γ2 subunit *(GABRG2)* gene in Japanese febrile seizure patients. Neurosci Lett. 2003;343:117–20. 12759178 10.1016/s0304-3940(03)00338-0

[31] Kang J, Shen W, Macdonald RL. Trafficking-deficient mutant *GABRG2* subunit amount may modify epilepsy phenotype. Ann Neurol. 2013;74:547–59. 23720301 10.1002/ana.23947 PMC3839255

[32] Macdonald RL, Kang J, Gallagher MJ. Mutations in GABAA receptor subunits associated with genetic epilepsies. J Physiol. 2010;588:1861–9. 20308251 10.1113/jphysiol.2010.186999 PMC2901974

[33] Pérez-Pérez D, Frías-Soria CL, Rocha L. Drug-resistant epilepsy: from multiple hypotheses to an integral explanation using preclinical resources. Epilepsy Behav. 2021;121:106430. 31378558 10.1016/j.yebeh.2019.07.031

[34] Kwan P, Schachter SC, Brodie MJ. Drug-resistant epilepsy. N Engl J Med. 2011;365:919–26. 21899452 10.1056/NEJMra1004418

